# Promising Antineoplastic Actions of Melatonin

**DOI:** 10.3389/fphar.2018.01086

**Published:** 2018-10-16

**Authors:** Gaia Favero, Enrico Moretti, Francesca Bonomini, Russel J. Reiter, Luigi Fabrizio Rodella, Rita Rezzani

**Affiliations:** ^1^Anatomy and Physiopathology Division, Department of Clinical and Experimental Sciences, University of Brescia, Brescia, Italy; ^2^Interdipartimental University Center of Research “Adaption and Regeneration of Tissues and Organs,” University of Brescia, Brescia, Italy; ^3^Department of Cell Systems and Anatomy, UT Health Science Center, San Antonio, TX, United States

**Keywords:** apoptosis, cancer hallmarks, cancer treatment, melatonin, oncology

## Abstract

Melatonin is an endogenous indoleamine with an incredible variety of properties and activities. In recent years, an increasing number of studies have investigated this indoleamine’s interaction with cancerous cells. In particular, it seems that melatonin not only has the ability to improve the efficacy of many drugs used in chemotherapy but also has a direct inhibitory action on neoplastic cells. Many publications underlined the ability of melatonin to suppress the proliferation of various cancer cells or to modulate the expression of membrane receptors on these cells, thereby reducing tumor aggressiveness to metastasize. In addition, while melatonin has antiapoptotic actions in normal cells, in many cancer cells it has proapoptotic effects; these dichotomous actions have gained the interest of researchers. The increasing focus on melatonin in the field of oncology and the growing number of studies on this topic require a deep understanding of what we already know about the antineoplastic actions of melatonin. This information would be of value for potential use of melatonin against neoplastic diseases.

## Introduction

Globally, cancer is the second leading cause of death in the order of incidence, next only to cardiovascular diseases (Ferlay et al., 2012; Bray and Soerjomataram, 2015; Fitzmaurice et al., 2015). In 2012, 8.2 million cancer deaths and 14.1 million new cases of cancer occurred worldwide, as estimated in the GLOBOCAN study (Ferlay et al., 2015). Lung cancer causes the highest incidence of number of deaths in both males and females, whereas prostate cancer is the first in incidence in male patients and breast cancer is the first in incidence in female patients (Ferlay et al., 2012; Fan et al., 2015; Torre et al., 2016; James et al., 2017).

**FIGURE 1:**
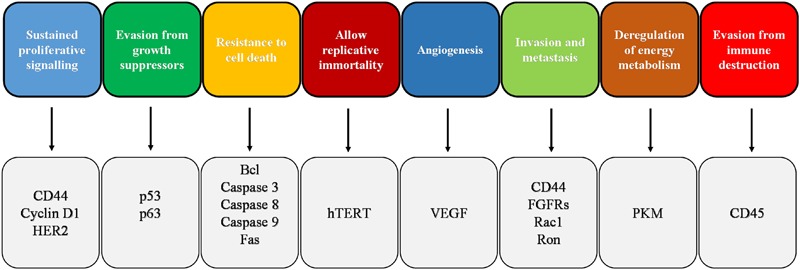
Schematic representation of cancer hallmarks with the indication of the main markers involved in the corresponding hallmark. FGFR, fibroblast growth factor receptor; HER2, human epidermal growth factor receptor 2; PKM, pyruvate kinase M; VEGF, vascular endothelial growth factor.

Hanahan and Weinberg (2000) identified six fundamental biological processes called hallmarks that permit the initiation and growth of cancer: sustained proliferative signaling, evasion from growth suppressors, resistance to cell death, allow replicative immortality, angiogenesis, and invasion and metastasis. With subsequent progress in cancer research and the development of new scientific evidence, reprogramming of energy metabolism and evasion from immune destruction were added to the previous list of cancer hallmarks (Hanahan and Weinberg, 2011; **Figure 1**). In the following paragraphs, we review briefly the effect of melatonin on the main cancer hallmarks and, thereafter, we focus our attention on melatonin induction of cancer cell death by apoptosis.

**FIGURE 2:**
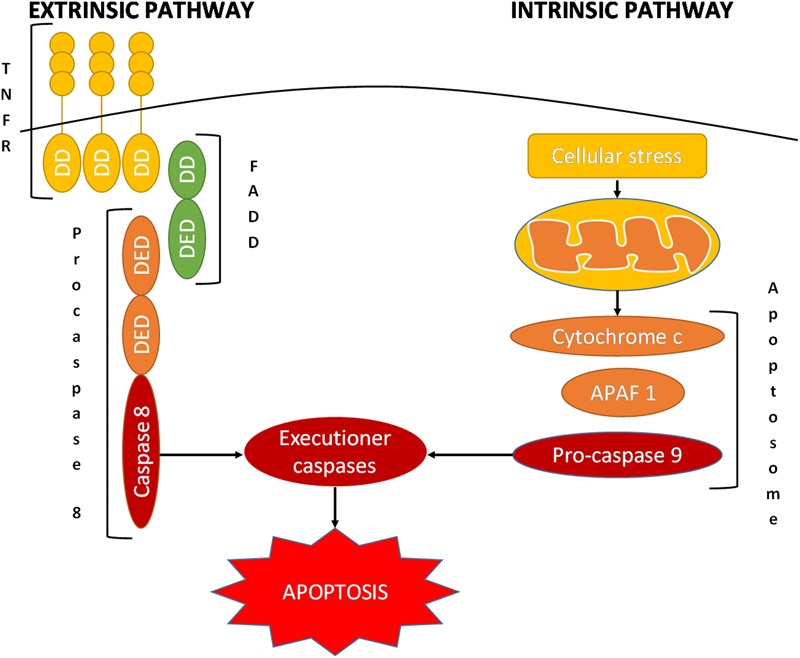
Schematic representation of the extrinsic and intrinsic pathways of apoptosis. APAF 1, apoptotic protease-activating factor 1; DD, death domain; DED, death effector domain; FADD, Fas-associated protein with death domain; TNFRSF, tumor necrosis factor receptor superfamily.

To date, many strategies and targets have been identified and studied in the battle against cancer. One of the potentially valuable mechanisms to reduce cancer is the induction of apoptosis of the cancerous cells, an endogenous mechanism that induces the cell to self-destruct (Derakhshan et al., 2017; Rathore et al., 2017). Apoptosis is an evolutionary highly conserved mechanism of programmed cell death that plays a critical role in homeostasis as well as in the development of tissues (Ricci and Zong, 2006; Fulda, 2017). A large variety of stimuli can induce apoptosis through the finely regulated activity of various proteins and complexes that eliminate any unnecessary cell through extrinsic or intrinsic pathways of apoptosis (Dasgupta et al., 2016; Matsuura et al., 2016; Fulda, 2017; Larsen and Sørensen, 2017; Pfeffer and Singh, 2018). A key role in these processes is played by caspases that function as cysteine proteases (Hassan et al., 2014; Fulda, 2017). In each apoptotic pathway, there is an initiator caspase, i.e., caspase 8 in the extrinsic pathway and caspase 9 in the intrinsic pathway; the action of these enzymes lead to the activation of executioner caspases, i.e., caspase 3, 6, or 7 (Matsuura et al., 2016; **Figure 2**). Under the proteolytic activity of caspases, the cell undergoes destruction of organelles, degradation of mitochondrial RNAs, and marked morphological changes, resulting in complete cell fragmentation (Ricci and Zong, 2006; Larsen and Sørensen, 2017; Pfeffer and Singh, 2018).

The extrinsic pathway activates apoptosis via the interaction of trimeric death receptors of the tumor necrosis factor receptor superfamily (TNFRSF) with their ligands (González-Flores et al., 2014; Seol et al., 2015; Vanamee and Faustman, 2018). Some of these receptors include the fast apoptotic signal receptor (Fas), TNFα-related apoptosis-inducing ligand-receptor 1 (TRAIL-R1), and TRAIL-R2 (Fulda, 2013; Gieffers et al., 2013). As these receptors activate their intracellular domain, referred to as the death domain (DD), they bind the adaptor Fas-associated death domain protein (FADD); this is composed of a DD itself and a death effector domain (DED). This last component converts procaspase 8 into its active form, caspase 8, which in turn activates the executioner caspases (Chuffa et al., 2016; Fulda, 2017; Miles and Hawkins, 2017; Herr, 2018).

In contrast, the intrinsic pathway is initiated by cellular stress, which induces the mitochondria to release cytochrome c into the cytosol (Miles and Hawkins, 2017; Reiter et al., 2017a; Thangarajan et al., 2018). Cytochrome c binds apoptotic protease-activating factor 1 (APAF 1), which recruits procaspase 9 via the interaction of caspase activation and recruitment domains (CARDs) (Ricci and Zong, 2006). These proteins together form the apoptosome, executioner caspase-activating complex, leading to the caspase cascade and hence programmed cell death (Ricci and Zong, 2006; Matsuura et al., 2016; Pfeffer and Singh, 2018).

Considering also the knowledge about the molecules involved in apoptosis, we should be able to exploit new target structures for oncologic therapies and for a better understanding of some actions of antitumor drugs already in use. In fact, self-destruction of neoplastic cells is one of the main means by which an organism prevents the initiation of cancer (Fernald and Kurokawa, 2013; Dasgupta et al., 2016; Pfeffer and Singh, 2018). When the apoptotic control is lost, the mutated cell evades the first chance of negative selection. If not stopped, the cancer grows in size and accumulates mutations thereby dysregulating proliferation, differentiation, and angiogenesis (Hassan et al., 2014; Ocaña et al., 2018; Tucci et al., 2018).

A variety of different “classical” therapeutic strategies have been used to eliminate cancer or stop its development. For many cancer types, such as some hematologic tumors, chemotherapy is the most effective treatment (Mousavi et al., 2016; Bell et al., 2018). For other types, surgery is the best option (Marcasciano et al., 2017; Ye et al., 2017); when surgery is not possible, chemotherapy can be helpful in stopping tumor growth or reducing its volume (Wagner et al., 2017; Wang et al., 2018). Unfortunately, in many cases antitumor drugs are not effective and they often have deleterious side effects (Iwamoto et al., 2014; Li and Caeyenberghs, 2018). Most cancer biologists agree that there is a need for new antineoplastic therapies or cotherapies, and proapoptotic strategies seem to be promising against neoplastic diseases (Chesi et al., 2016; Perimenis et al., 2016; Rathore et al., 2017).

It is important to underline that some “classical” oncologic therapies are already proapoptotic molecules/drugs (Zhao et al., 2014; Li et al., 2015; Derakhshan et al., 2017; Fujita et al., 2017). For example, drugs from the family of proteasome inhibitors, such as Bortezomib, prevent the normal degradation of cyclin proteins and progression of the cell cycle, thus inducing apoptosis in target cells (Wu and Shi, 2013; Vriend and Reiter, 2014). Furthermore, many other studies have tested the proapoptotic activity of different molecules with the intent of finding new clinical drugs for chemotherapy. Thus, various studies have been conducted to understand the effect of different death receptor ligands. Agonistic antibodies for TRAIL-R1 and -R2 have not only controversial effects due to their antineoplastic activity but also potentially proneoplastic effects depending on the context (Chen et al., 2013; Fulda, 2013; Micheau et al., 2013). Even so, some recent TRAIL-R agonists possess a better antitumor efficacy due to their improved clustering ability on the target receptors (Gieffers et al., 2013). Other possible targets are Bcl-2 family components, regulators of the mitochondrial pathway of apoptosis (Popgeorgiev et al., 2018), and the promoters of caspase 8 hypermethylation in those cancer cells that have its expression suppressed, as described in some hepatocellular carcinoma mouse models (Fulda et al., 2001; Liedtke et al., 2005; Fulda, 2017).

Due to also its emerging proapoptotic effects, melatonin is being heavily investigated as a potential antineoplastic adjuvant (Rodriguez et al., 2013; Guven et al., 2016; Reiter et al., 2017a; Waseem et al., 2017; Talib, 2018). Various studies also on its proapoptotic properties in neoplastic diseases suggest that it may be considered a promising molecule for “tomorrow’s chemotherapy” (Vriend and Reiter, 2014; Fan et al., 2015; Chovancova et al., 2017; Gatti et al., 2017).

## Melatonin

Melatonin, or N-acetyl-5-methoxytryptamine, is an indoleamine synthesized from tryptophan by the pineal gland and perhaps all organs, since its production has been found to be associated with mitochondria (Venegas et al., 2012; Acuña-Castroviejo et al., 2014; Gandhi et al., 2015; Reiter et al., 2017b). In addition to being produced in all animals, melatonin also exists in plants (Reiter et al., 2013; Hardeland, 2016) as well as in plant derivatives, i.e., olive oil, wine, tomato, juices, and beer are some of the main dietary products where melatonin has been identified (Garcia-Moreno et al., 2013; Vitalini et al., 2013; Fernández-Pachón et al., 2014; Favero et al., 2017b).

**FIGURE 3:**
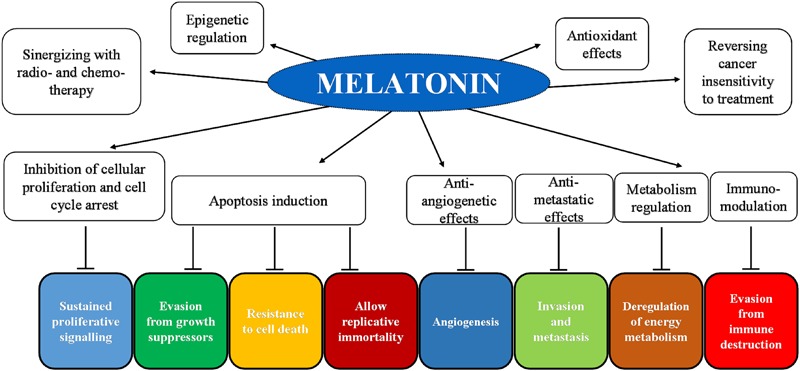
Schematic representation of melatonin’s inhibition of cancer hallmarks with an indication of the main target molecules and the corresponding hallmark.

Night-time darkness is a requirement for the pineal production of melatonin, which follows daily and seasonal patterns of secretion due to a light-sensitive retino-pineal pathway (Reiter, 1993; Stehle et al., 2011; Reiter et al., 2014; Trivedi and Kumar, 2014; Claustrat and Leston, 2015; Vriend and Reiter, 2015). Throughout life, melatonin levels change: nocturnal melatonin circulating levels are the highest in young children and decline in older people (Karasek and Reiter, 2002; Scholtens et al., 2016; Tan et al., 2018), although a great interindividual variability exists (Arendt, 1988; Scholtens et al., 2016). Notably, high melatonin levels are suggested to play positive and important roles in health and aging (Bubenik and Konturek, 2011; Venegas et al., 2012; Hardeland, 2013; Hill et al., 2013; Reiter et al., 2016; Scholtens et al., 2016; Majidinia et al., 2018). Currently, humans face a serious perturbation of the melatonin rhythm due to an altered light–dark cycle with the increased light pollution and with the majority of time spent indoors during the day, thereby causing a deregulation of the circadian system and of melatonin circulating levels (Reiter et al., 2006; Erren and Reiter, 2009). This condition, called chronodisruption (Erren and Reiter, 2009), is associated with epigenetic abnormalities and also with an increased incidence of metabolic, cardiovascular, neurologic, and also oncologic diseases (Reiter et al., 2006; Blask et al., 2011; Bonmati-Carrion et al., 2014; Cipolla-Neto et al., 2014; Haim and Zubidat, 2015; Peschke et al., 2015; Hardeland, 2017). Notably, melatonin exerts multiple actions by both receptor-dependent and receptor-independent mechanisms (Reppert et al., 1995; Slominski et al., 2012; Reiter et al., 2014). It is well established that melatonin possesses antioxidant and anti-inflammatory activities, and it influences the sleep–wake cycle, reproduction, and metabolism (Hardeland, 2005; Reiter et al., 2014; Hu et al., 2016; Favero et al., 2017a; Galano and Reiter, 2018). Hence, melatonin’s antioxidant activity is due not only to its ability to act as a scavenger agent but also to its capacity to upregulate antioxidant enzyme activity and downregulate prooxidant enzymes (Dominguez-Rodriguez et al., 2009; Halladin et al., 2014; Reiter et al., 2016). Interestingly, in the last decades, various studies investigated the effects of melatonin against cancer and identified its antiproliferative, cytostatic, antioxidant, cytotoxic, proapoptotic, and differentiative activities together with its ability to regulate epigenetic responses (Paternoster et al., 2009; Paroni et al., 2014; Haim and Zubidat, 2015; Liu et al., 2016; Ma et al., 2016; Mao et al., 2016; Chovancova et al., 2017; Li et al., 2017; Pariente et al., 2018; Posadzki et al., 2018; **Figure 3**).

### Melatonin and Antiproliferative and Cytostatic Actions on Cancer Cells

One of the effects of melatonin that makes it useful in antitumor therapy is its ability to reduce neoplastic proliferation involving both cytostatic and cytotoxic effects (Martín et al., 2007; Cabrera et al., 2010; Loureiro et al., 2015; Liu et al., 2016; Shen et al., 2016). In 2013, the study of Liu et al. documented the antiproliferative action of melatonin in a human MG-63 osteosarcoma cell line showing the downregulation of cyclin D1, cyclin B1, and cyclin-dependent kinases (CDK) 4 and 1; these are some of the principal regulatory components that determine cell cycle progression from a mitotic stage to the next (Liu and Cheng, 2013; Aleem and Arceci, 2015; Roskoski, 2016). Later, the same team investigated in depth the mechanisms by which melatonin downregulates these proteins and the growth of MG-63 osteosarcoma cells. Their results suggested that melatonin inhibits proliferative ERK1/2 signaling, pathway that controls gene expression and promotes cell cycle progression and cell division (Liu et al., 2016). Notably, melatonin activates ERK1/2 signaling in normal cells; on the contrary, it inhibits ERK1/2 in cancer cells, impeding their proliferation and potentially breaking their resistance to cytotoxic therapies (Asghari et al., 2018).

Many studies also describe the activity of melatonin against mammary cancer (Wang J. et al., 2012; Koşar et al., 2016; Gatti et al., 2017). A reduction in circulating melatonin leads to an increase in the incidence of mammary tumors induced by the carcinogen 7,12-dimethylbenz(a)-anthracene (DMBA), while melatonin treatment reduced the incidence (Proietti et al., 2013; Vriend and Reiter, 2015; Chu et al., 2018; Rybnikova and Portnov, 2018). In a breast cancer *in vitro* model, MCF-7 cells, melatonin has a cytostatic effect causing the accumulation of cells in the G0/G1 phase of the cell cycle or delaying the progression to the S phase of the cell cycle; similar results were observed also in T47D and ZR75-1, estrogen-sensitive breast cancer cell lines (Cos et al., 1996; Proietti et al., 2013, 2014; Nooshinfar et al., 2016). Melatonin inhibits MCF-7 cell growth according to a bell-shaped curve, showing that the highest cytostatic effect is generally obtained around the physiological range of supplementation (10^-11^–10^-9^ M). Higher or lower concentrations produce little or no tumor cell growth inhibition (Cos et al., 1991). However, in an anchorage-independent culture system, the dose-response curve becomes moderately linear and, increasing the melatonin concentration, there is a progressively greater cancer cells growth inhibition (Cos and Blask, 1990), underlining that cellular attachment to a substratum plays an important role in setting the level of cell sensitivity to melatonin (Proietti et al., 2013).

The addition of melatonin (400–800 μM for 24–72 h) to medium containing ovarian cancer cells OVCAR-429 showed a dose- and time-dependent reduction of cancer cell proliferation (Shen et al., 2016). The same study demonstrated that melatonin’s cytostatic effect induced an increase in the number of cells in the G1 phase but decreased those in the S phase (Shen et al., 2016). The hepatocarcinoma HepG2 cell line showed cycle arrest and apoptosis induced by the administration of melatonin (Martín-Renedo et al., 2008). Remarkably, in melanoma SK-MEL-1 cells, melatonin treatment caused a significant cytostatic effect rather than cytotoxic action, arresting tumor cells in the G1 phase of the cell cycle and thus reducing the neoplastic growth (Cabrera et al., 2010).

All these observations led to the conclusion that the inhibition of proliferation and the induction of cell cycle arrest are both strongly influenced by the accumulation of melatonin in cancer cells (Shen et al., 2016).

### Melatonin and Antimetastatic Effect

The major reason for most cancer deaths is tumor metastasis, which is possibly due to both reorganization of cancer cells’ gene expression and altered differentiation that lead to the epithelial-to-mesenchymal transition (EMT) (Ding, 2013; Bill and Christofori, 2015; Reiter et al., 2017a; Zhang et al., 2018). Altered cell-to-cell linkage permits the separation of neoplastic cells from the primary tumor and then the modifications of the extracellular matrix allow tumor cells to penetrate the surrounding stroma to reach the blood vessels, thereby generating metastasis (Cavallaro and Christofori, 2001; Langley and Fidler, 2011; Zheng et al., 2016). Owing to the broad range of melatonin’s properties, efforts to understand the oncostatic role of melatonin have recently shifted toward the process of tumor metastasis (Reiter et al., 2017a). In some types of cancer, it has been demonstrated that melatonin has also important antimetastatic effects specifically due its ability to prevent the EMT (Gonçalves Ndo et al., 2016; Lin et al., 2016; Mao et al., 2016; Akbarzadeh et al., 2017; Chen et al., 2017). Other antimetastatic mechanisms of melatonin include cytoskeletal reorganization (Ortíz-López et al., 2009), modulation of cell matrix (Hynes, 2002), and inhibition of angiogenesis (Lissoni, 2002; Su et al., 2017).

The mitogen activated-protein kinases/extracellular signal-regulated kinases (Mapk/Erk) signaling by the human epidermal growth factor receptor 2 (HER2) induces a rise in invasiveness and metastasis of human breast cancer cells (Spigel and Burstein, 2002). Administration of melatonin significantly reduces the activity of Mapk/Erk signaling (Mao et al., 2016). In the same study, athymic nude female mice were implanted with breast cancer cells to form tumor xenografts and significantly fewer metastatic foci in the lungs of melatonin-treated mice were observed (6 to 13 metastatic lung foci) (Mao et al., 2016). Borin et al. (2016) observed similar results *in vitro* human breast cancer cell lines MDA-MB-231 (metastatic, ERα-negative). Furthermore, melatonin has been shown to convert the human breast cancer cell line MCF-7 to a less invasive phenotype by increasing expression of E-cadherin, a prototypical member of the type-1 classical cadherins whose loss favors tumor metastasis (Cos et al., 1998; Chuffa et al., 2017). The anti-invasive effect of melatonin on breast cancer may be also through the downregulation of the p38 pathway and suppression of metalloproteinases-2 and -9 expression and activity (Mao et al., 2010). In addition, melatonin exhibited both antiproliferative and proapoptotic effects in the metastatic breast cancer cell line MDA-MB-361, through the activation of the APAF 1/caspase-dependent apoptotic pathway (Wang J. et al., 2012). In particular, Wang J. et al. (2012) observed that melatonin, in a dose-dependent manner, induced APAF 1 expression that forms a complex with caspase 9 in the presence of cytochrome c and dATP, ultimately, leading to caspase 9 and caspase 3 activation and subsequently inducing apoptosis.

Induced hypoxia in glioma U251 and SWO-38 cell lines reduces the expression of E-cadherin and α-catenin, while it induces mesenchymal markers, including N-cadherin, vimentin, and SNAIL1, which normally promote the EMT during embryonic development (Chen et al., 2017). In U251 and SWO-38 cell lines, melatonin suppresses cell migration and invasion induced by hypoxia (Chen et al., 2017; Su et al., 2017). In the human ovarian cancer SKOV3 cell line, melatonin decreased the expression of EMT-related genes, such as SNAIL and vimentin, while it increased E-cadherin expression (Akbarzadeh et al., 2017).

The vast amount of scientific evidence related to melatonin’s antimetastatic activity prompts the conclusion that melatonin may be an useful adjuvant to prevent cancer dissemination and metastasis.

### Melatonin and Its Antineoplastic Proapoptotic Action

**FIGURE 4:**
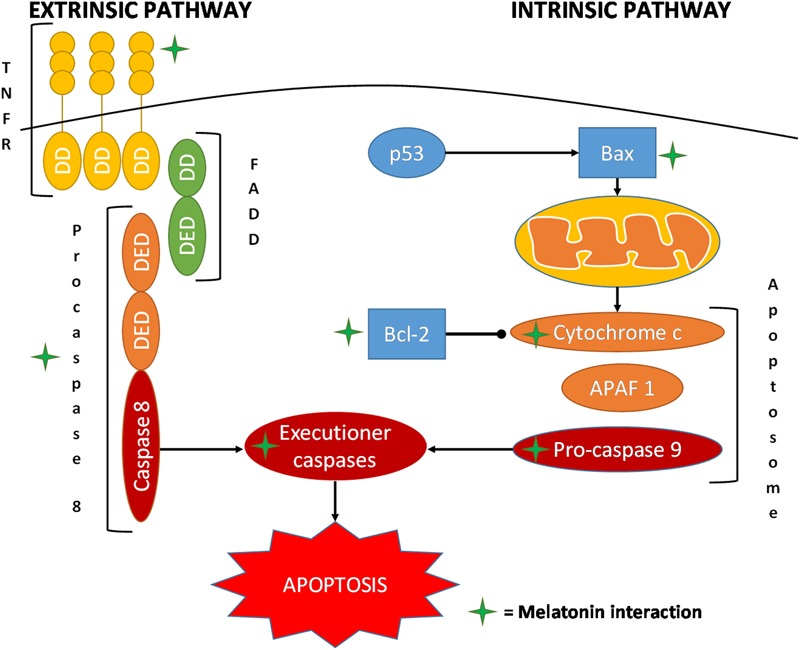
Schematic representation of the main interaction of melatonin in the pathways of apoptosis. APAF 1, apoptotic protease-activating factor 1; DD, death domain; DED, death effector domain; FADD, Fas-associated protein with death domain; TNFRSF, tumor necrosis factor receptor superfamily.

An interesting aspect of melatonin’s antitumor effect is its capacity of inducing apoptosis, a response only observed in cancer cells; this leads to an effective reduction in cancer volume, thus improving the clinical condition of the patient (Seely et al., 2012; Chovancova et al., 2017; Fulda, 2017; Talib, 2018; **Figure 4**).

Melatonin treatment (10 μM) for 24 h of the colorectal carcinoma cell line DLD1 or the ovarian cancer cell line A2780 doubled the apoptotic events compared to that observed in normal control cells. The use of specific siRNAs illustrated the role of type 1 inositol triphosphate receptor and type 1 sodium/calcium exchanger in mediating this proapoptotic action of melatonin (Chovancova et al., 2017). In another study on ovarian cancer in rats, it was observed that melatonin, upregulating the cleaved caspase 3, p53, and Bax and downregulating Bcl-2, promoted apoptosis (Chuffa et al., 2016).

An *in vitro* study on breast cancer cells indicated that melatonin’s proapoptotic activity was accompanied by the induction of APAF 1 expression, demonstrating a significant reduction in melatonin-induced apoptosis after pretreatment with a specific siRNA (Wang J. et al., 2012). In breast cancer, melatonin was also observed to trigger cytochrome c release and stimulate caspase 3 and 9 activities and cleavage (Wang J. et al., 2012). In the nanomolar range, melatonin increases the p53 to phosphorylated mouse double minute 2 homolog (MDM2p) ratio and downregulates sirtuin1 (Bizzarri et al., 2013). In particular, treatment with melatonin of human MCF-7 breast cancer cells induced a significant reduction in levels of MDM2p, the major physiological antagonist of p53 (Cucina et al., 2009; Proietti et al., 2011). The reduced MDM2p levels allowed p53 to evade this control and stimulated apoptotic cellular death (Bizzarri et al., 2013).

Interestingly, in leukemia Molt-3 cells, incubation with melatonin induced apoptotic death through a caspase-dependent mechanism (Perdomo et al., 2013). In fact, in this study it was observed that caspase 3, 9, 6, and 7 were activated but not caspase 8 and 2. Melatonin also upregulated the proapoptotic factor Bax and increased the release of cytochrome c (Perdomo et al., 2013; Fulda, 2017). Pretreatment with z-VAD-fmk, a general caspase inhibitor, significantly reduced melatonin-induced apoptosis (Perdomo et al., 2013; Fransolet et al., 2015). Similar results were obtained in the human HL-60 myeloid cell line, showing, after 1 mM melatonin treatment, an increase of apoptosis and a slight rise in necrosis (Rubio et al., 2007; Bejarano et al., 2011). These results were associated with a significant elevation in caspase 3 and 9 activities, depolarization of the mitochondrial membrane, and activation of its transition pores. These effects were time-dependent, reaching the maximum value at 12 h (Bejarano et al., 2009).

The interaction of melatonin with hematological cancers has been extensively investigated. Casado-Zapico et al. (2011) performed an extensive study on lymphoma, acute lymphoid leukemia, and chronic and acute myeloid leukemia. The results confirmed the increase in melatonin-dependent apoptosis in the examined cancers, showing a pronounced rise of caspase 8 and of the proapoptotic protein Bid. Furthermore, the extrinsic apoptotic pathway was involved due to the augmented expression of both Fas and its ligand FasL (Casado-Zapico et al., 2011; Bizzarri et al., 2013).

Treatment of cultured human lung adenocarcinoma cells with melatonin resulted in the increase of caspase 3 activity, upregulation of Bax and p53, and downregulation of Bcl-2 (Fan et al., 2015).

In SW-1990, a pancreatic cancer cell line, the proapoptotic effect of melatonin was investigated using the annexin V/propidium iodide assay, to distinguish necrotic and apoptotic cells; it was found that both necrosis and apoptosis increased in a dose-dependent manner. Furthermore, western blot and RT-PCR evaluation of Bax and Bcl-2 expressions showed that they were up- and downregulated, respectively (Xu et al., 2013). In human pancreatic carcinoma cells, an upregulation of caspase 9 was seen; while the levels of Bax increased, the cytoplasmic levels of Bcl-2 did not drop. Both proteins were upregulated, but with a significant reduction of the Bcl-2 to Bax ratio; this was most evident with the administration of the physiological concentration of melatonin (10^-12^ M) compared to higher concentrations (10^-8^ and 10^-10^ M) (Leja-Szpak et al., 2010). Similar results were obtained in studies on prostatic cancer or hepatic cancer with the activation of caspase 3 and 9 and the induction of cancer cell apoptosis (Martín-Renedo et al., 2008; Kim and Yoo, 2010).

Gatti et al. (2017) used four different melatonin derivatives to study their effects on *in vitro* melanoma and breast cancer cells: UCM 976, UCM 1032, UCM 1033, and UCM 1037. The most promising molecule, UCM 1037, showed a proapoptotic action on melanoma cells and MDA-MB231 breast cancer cells, but in MCF-7 breast cancer cells it induced necrosis. Even if apoptosis was induced in melanoma and MDA-MB231 cells, no alteration of Bax levels was detected. On the contrary, cleaved caspase 3 was observed, indicating a caspase cascade-mediated activation of apoptosis. Interestingly, the WM-115 melanoma cancer cell line was an exception, showing no cleavage of caspase 3 but an inhibition of the antiapoptotic protein Bcl-2, which was not observed in the other cell lines (Gatti et al., 2017). These results led to the conclusion that the effect of the melatonin derivative UCM 1037 depends on the type of cancer cell and it may be possible that the same goes for melatonin.

Interestingly, it is generally assumed that melatonin at physiological concentrations could mainly exert a cytostatic action; meanwhile, apoptotic effects are often observed at higher concentrations (Blask et al., 2002; Bizzarri et al., 2013).

### Melatonin and Antineoplastic Palliative Effect

Another important beneficial action of melatonin is its ability to reduce chemotherapy and radiotherapy-induced toxicity and oxidative stress. Melatonin has also been reported to be useful in the treatment of the associated insomnia, cachexia, delirium, and other symptoms that often occur in cancer patients (Mahmoud et al., 2005; Davis and Goforth, 2014; da Silva et al., 2015; Bush et al., 2016; Waldman et al., 2016). There are also some studies that showed that melatonin has weak or no antineoplastic palliative effect (Del Fabbro et al., 2013; Lund Rasmussen et al., 2015). The meta-analysis published by Seely et al. (2012) documented the ability of melatonin to reduce the occurrence of alopecia, anemia, asthenia, and thrombocytopenia, which pooled the relative risk of 0.86, 0.83, 0.44, and 0.21, respectively. To investigate melatonin’s ability to attenuate anorexia, weight loss, and fatigue in patients with cancer, Del Fabbro et al. (2013) performed a randomized and double-blind 28-day trial in which 20 mg/day of melatonin was administrated at night in 48 patients with metastatic or local recurrent gastrointestinal or lung carcinoma. From baseline to day 28 there were no significant differences between melatonin and placebo groups regarding appetite, body weight, toxicity, or survival. There were also no significant differences between the two groups in the symptoms. Patients with histologically confirmed tumor-node-metastasis stage IV who felt significantly tired were recruited for a randomized, double-blind 2 weeks trial in which 20 mg/day of melatonin was administered to investigate the effect of melatonin on fatigue and other symptoms that negatively impact the quality of life of cancer patients. The results did not show any significant difference between the investigated groups regarding physical fatigue or secondary outcomes (Lund Rasmussen et al., 2015).

### Melatonin in Association With Chemotherapy

Many studies have tested the efficacy of melatonin as an antitumor therapy together with chemotherapy in lung, breast, cervical, colon, hepatic, hematological, and other cancer types (Koşar et al., 2016; Lu et al., 2016; Quintana et al., 2016; Chen et al., 2017; Hao et al., 2017; Pariente et al., 2017). A meta-analysis performed by Wang Y. M. et al. (2012) considered eight randomized controlled trials related to the use of melatonin in solid tumor therapy. In these trials, 20 mg of melatonin was given daily together with chemotherapy, resulting in complete or partial remission of the tumors (16.5 vs. 32.6%), significant improvement of 1-year survival rate (28.4 vs. 52.2%), and reduction of the radio-chemotherapy side effects, including thrombocytopenia, neurotoxicity, and fatigue. In human colorectal adenocarcinoma HT-29 cells, incubation with 1 mM melatonin increased the cytotoxic effects of 5-fluorouracil (5-FU), raising the population of cancer apoptotic cells (Gao et al., 2017; Pariente et al., 2018). Interestingly, melatonin further strengthened the effects of 5-FU but did not significantly sensitize colorectal cancer cells to cisplatin-driven cell cycle arrest. These diverse effects can be explained on the basis of the different mechanisms of action. Indeed, cisplatin predominantly exerted a cytostatic action that led to a reduction in cell proliferation over time, whereas 5-FU triggered reactive oxygen species-dependent apoptosis, resulting in a drastic decrease in cell viability (Pariente et al., 2017, 2018). Likewise, melatonin has been proven to attenuate antitumor actions of cisplatin in human liver carcinoma HepG2 cells via a counter-balance between the roles of apoptotic- and autophagy-related proteins (Bennukul et al., 2014; Bonomini et al., 2018). Despite this, it has been observed that melatonin enhances cisplatin-induced cytotoxicity in different human ovarian cancer cells, like SKOV3, HTOA, and OVCAR-3 (Futagami et al., 2001; Kim et al., 2012; Pariente et al., 2017). Furthermore, in a human hepatocellular carcinoma cell line, Bel-7402, the combination of cisplatin and melatonin reduced the IC50 value of cisplatin and increased cisplatin-induced apoptosis (Moreira et al., 2015; Hao et al., 2017); similar results were also obtained in a human cervical cancer cell line, HeLa (Chen et al., 2017).

Notably, the combination of doxorubicin and melatonin showed a better efficacy than doxorubicin alone in inducing apoptosis, mitochondrial membrane depolarization, and caspase 3 and 9 activation in MCF-7 human breast cancer cells (Koşar et al., 2016). On the drug-resistant MCF-7 cells, modest growth inhibition was achieved by melatonin supplementation at the concentrations of 80 and 2000 pg/mL, while the cytotoxicity of doxorubicin was significantly increased following treatment with 100 pg/mL of melatonin. Additionally, 40–80 pg/mL of melatonin reduced the growth of P388 mouse leukemia cells with no enhancement of doxorubicin cytotoxicity, on drug-resistant P388 cells, melatonin alone significantly reduced cell growth at 400–1000 pg/mL and displayed, at 100–200 pg/mL, a pronounced dose-dependent enhancement of doxorubicin cytotoxicity (Granzotto et al., 2001; Asghari et al., 2018). The cytotoxicity of melatonin has generally been observed in cancer cells treated with high millimolar concentrations of this indoleamine (Bizzarri et al., 2013; Pariente et al., 2017).

The ability of melatonin to selectively sensitize cancer cells to cytotoxic therapies, while protecting normal cells from toxicities of such agents, justifies its consideration as a potential adjuvant to cancer treatment and also encourages further research in this field.

### Melatonin and Epigenetic Alterations in Cancer Cells

As epigenetic modifications are involved in the pathogenesis of several neoplastic diseases, including prostate, gastric, lung, and breast cancers (Jerónimo et al., 2011; Nowsheen et al., 2014; Haim and Zubidat, 2015; Hong, 2018; Liao and Xu, 2018), it is important to underline that melatonin may also regulate epigenetic responses (Korkmaz and Reiter, 2008; Haim and Zubidat, 2015; Pan and Niles, 2015; Li et al., 2017). Epigenetic mechanisms involve activation of oncogenes and deactivation of cancer suppressor genes, and the gene expression in both cases is affected by chromatin remodeling of its binding sites of transcriptional factors (Choi and Lee, 2013; Riley, 2018). To date, the melatonin modulation of epigenetic responses is not completely established. However, melatonin can regulate epigenetic modifications in cancer cells by both DNA methylation and histone protein remodeling (Haim and Zubidat, 2015). In human breast cancer cell lines, melatonin increased DNA methylation and induced downregulation of the oncogenes EGR3 and POU4F2/Brn-3b and upregulation of the tumor suppressor gene GPC3 (Lee et al., 2013; Li et al., 2017). Melatonin treatment can suppress human breast cancer cell proliferation by deacetylation of oncogenes, resulting in chromatin closing and thus in the inhibition of the binding of the transcriptional factor required for triggering the expression of oncogenes (Wang J. et al., 2012; Wang Y. M. et al., 2012; Haim and Zubidat, 2015). Furthermore, Pan and Niles (2015) observed that melatonin supplementation (0.5, 1, 10, and 100 nM) in human neuroblastoma SH-SY5Y cells induced histone hyperacetylation/chromatin remodeling on gene transcription through the G protein-coupled melatonin receptor MT1. Interestingly, melatonin can provide the missing link between the environmental disruption of biological rhythms and the epigenetic molecular machinery that regulates global DNA hypomethylation in oncogenes and local DNA hypermethylation in tumor suppressor genes. Although substantial and significant progress has been made in understanding the molecular basis of epigenetic-induced tumorigenesis, the exact relation among circadian disruption, melatonin and aberrant DNA methylation and histone acetylation requires further research.

## Conclusion

Melatonin possesses an incredible variety of actions and one of the most promising is its antineoplastic effect. In particular, melatonin inhibits more than one of the cancer hallmarks due to its antiproliferative, cytostatic, antimetastatic, and proapoptotic effects against tumor cells. Furthermore, melatonin might regulate epigenetic responses and this was found useful when combined with various chemotherapy drugs, increasing their efficacy with synergic interactions and also reducing their collateral side effects. Another important and promising aspect of melatonin against cancer is its ability to induce neoplastic cells to self-destruct. Currently, melatonin seems able to improve the clinical outcome of patients afflicted by cancer. Although numerous studies have shown the ability of melatonin to induce the death of cancer cells by apoptosis, further studies to consider melatonin as a sole antineoplastic therapy and to clarify its mechanisms of action are essential. However, melatonin should be considered, minimally, as a cotherapy to be used together with “classical” chemotherapies. The rising number of clinical trials on melatonin against cancer will permit a better understanding of its mechanisms of action, and in which types of cancer and with which drugs it should or should not be used. Research related to melatonin and cancer has progressed very rapidly with new discoveries being made. This brief review is meant to be an introduction to melatonin’s multiple actions in limiting cancer growth.

## Author Contributions

GF and EM critically analyzed the bibliography. GF, EM, and FB wrote the manuscript and drew the figures. LFR and RR conceived the idea. RJR extensive English and editing revision. LFR, RJR, and RR improved the manuscript content. GF and EM contributed equally to this work. All authors agreed on the finally submitted version of the manuscript.

## Conflict of Interest Statement

The authors declare that the research was conducted in the absence of any commercial or financial relationships that could be construed as a potential conflict of interest.
